# Cellular Aspects of *Shigella* Pathogenesis: Focus on the Manipulation of Host Cell Processes

**DOI:** 10.3389/fcimb.2016.00038

**Published:** 2016-03-31

**Authors:** Samuel A. Killackey, Matthew T. Sorbara, Stephen E. Girardin

**Affiliations:** ^1^Department of Laboratory Medicine and Pathobiology, University of TorontoToronto, ON, Canada; ^2^Department of Immunology, University of TorontoToronto, ON, Canada

**Keywords:** *Shigella*, innate immunity, bacterial infections, Nod1 signaling adaptor protein, Nod2 signaling adaptor protein, toll-like receptors, NLR, autophagy

## Abstract

*Shigella* is a Gram-negative bacterium that is responsible for shigellosis. Over the years, the study of *Shigella* has provided a greater understanding of how the host responds to bacterial infection, and how bacteria have evolved to effectively counter the host defenses. In this review, we provide an update on some of the most recent advances in our understanding of pivotal processes associated with *Shigella* infection, including the invasion into host cells, the metabolic changes that occur within the bacterium and the infected cell, cell-to-cell spread mechanisms, autophagy and membrane trafficking, inflammatory signaling and cell death. This recent progress sheds a new light into the mechanisms underlying *Shigella* pathogenesis, and also more generally provides deeper understanding of the complex interplay between host cells and bacterial pathogens in general.

## Introduction

Bacteria of the genus *Shigella* are human pathogens that infect the gastro-intestinal tract and cause acute shigellosis. *Shigella*e are Gram-negative, non-motile, facultative anaerobic pathogens that are closely related to *Escherichia coli* but have evolved specific traits of pathogenicity, physiology and serology (Ud-Din and Wahid, 2014). *Shigella* infection can occur by the fecal-oral route of transmission, person-to-person contact or ingestion of contaminated food or water. There are four serogroups of *Shigella*: *Shigella flexneri, S. dysenteriae, S. boydii*, and *S. sonnei*, all of which are able to cause disease in humans (Livio et al., 2014). Diarrhea is an early symptom of shigellosis and may be initiated as the bacteria reach the small intestine. However, the bacteria predominantly target the colonic epithelium that they rapidly invade, causing inflammatory colitis (Ashida et al., 2015). According to data from the Centers for Disease Control (CDC) and Prevention, *Shigella* is estimated to cause 80–165 million cases worldwide every year, resulting in 0.6 million deaths, particularly in young children. *Shigella* spp. are endemic in a number of tropical and sub-tropical regions of the world where *S*. *flexneri* is the most common cause of disease, while *S. sonnei* is more frequently associated with infection in industrialized countries (Liang et al., 2007). Infection with *S. boydii* and *S. dysenteriae* are less common overall but can be locally endemic, such as in South Asia and in Sub-Saharian Africa (Kotloff et al., 2013).

*Shigella* is a strict human pathogen, and therefore animal models of infection have been difficult to establish, and only recapitulate some aspects of pathogenicity. Nonetheless, several animal models have been developed that include the rabbit ligated ileal loop model, the newborn mouse enteric infection model and the guinea pig enteric infection model (Perdomo et al., 1994; Fernandez et al., 2003; Shim et al., 2007). Recently, a new model of infection in the Zebrafish larvae was developed, which allowed study of the interaction between *Shigella* and phagocytes *in vivo* (Mostowy et al., 2013).

While studying the mechanisms of *Shigella* pathogenesis *in vivo* has proven difficult, *Shigella* infection, in particular using the *S. flexneri* species, has become one of the most widely used paradigms of host-bacterial interaction in cellular models of infection. Together with *Mycobacteria, Salmonella* and *Listeria, Shigella* represents one of the most studied bacteria that can invade (i.e., cross the host plasma membrane) host cells. Among those bacteria, the invasion mechanism triggered by *Shigella* has similarities to the one induced by the other Gram-negative bacterium, *Salmonella*; however, in contrast to *Salmonella* and *Mycobacteria, Shigella* rapidly escapes the entry vacuole, moves freely in the host cytosol, and is able to spread from cell to cell, which are properties shared with the Gram-positive bacterium *Listeria*. Thus, host cell invasion by *Shigella* has overall unique characteristics, and the use of this bacterium as a model of host-bacteria interaction over the past four decades has considerably increased our understanding of bacterial pathogenesis. In this review, we will provide an overview of some of the most recent progress that was made in cellular microbiology and innate immunity, using *Shigella* as a model.

## *Shigella* invasion

Strikingly, the inoculum size necessary for *Shigella* infection is as low as 100 bacteria (DuPont et al., 1989). In order to establish a productive infection, *Shigella* transits across the colonic epithelial layer through M cells, and is then able to efficiently invade colonic epithelial cells from the basolateral face (Phalipon and Sansonetti, 2007). Invasion of the colonic epithelium and spread from cell-to-cell is the primary driver of the severe inflammatory response associated with infection.

**Figure 1:**
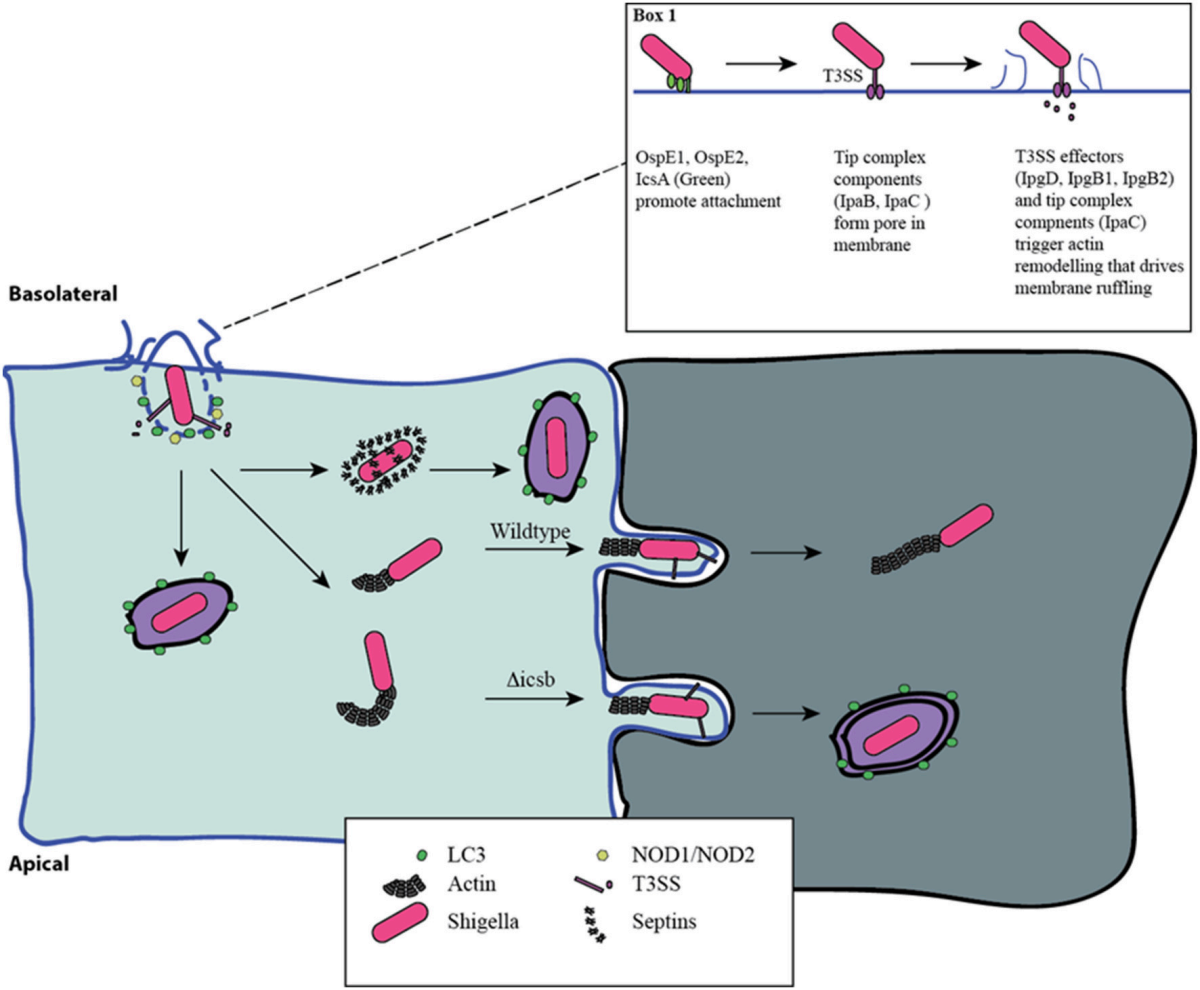
**Distinct phases of invasion and autophagy targeting during *Shigella infection***. *Shigella* adheres to the basolateral surface of epithelial cells, forms a pore in the eukaryotic membrane, and delivers effector proteins to induce its uptake (Box 1). The first wave of autophagy targeting follows initial invasion and is mediated by recruitment of the autophagy machinery to the site of entry. The intracellular PRRs, NOD1 and NOD2, play a critical role through the recruitment of ATG16L1. Following escape from the entry vacuole, *Shigella* drive actin polymerization at one pole through IcsA-dependent recruitment of N-WASP and ARP2/3. This allows for intracellular motility. This process is countered by the host's attempt to trap bacteria in septin-derived cages that enables autophagy targeting. Once motile and free in the cytosol the host is unable to target *Shigella* to autophagy. Actin-based motility allows *Shigella* to spread from cell-cell, and it efficiently escapes into the second cell using a reactivated T3SS. This secondary invasion event allows for additional autophagy targeting. IcsB mutants that are less efficient at escape are more readily targeted by autophagy at this step.

*Shigella* triggers its own uptake into epithelial cells using a type III secretion system (T3SS) (Figure 1). The proteins of the T3SS are encoded by a large 220 kb virulence plasmid and form a macromolecular needle-like structure that allows for the delivery of effector proteins across the membrane of the target eukaryotic cell. Prior to delivery of effectors, *Shigella first* adheres to the host cell, despite the absence of classical adhesion proteins. Recent work has demonstrated that the *Shigella* surface protein, IcsA, functions as an adhesin that is activated by bile-salts, and facilitates interaction with host cells after initial activation of the T3SS (Brotcke Zumsteg et al., 2014). Bile-salts also promote the secretion of OspE1 and OspE2 which remain on the bacterial outer-membrane and increase adherence to polarized cells (Faherty et al., 2012). In addition, bile-salts, in particular deoxycholate, promote final assembly of the T3SS in an activation-ready state (Stensrud et al., 2008). Furthermore, bacterial binding to filopodia through the T3SS components, IpaB and IpaD, also promotes interaction and invasion (Romero et al., 2011). Interestingly, Marteyn et al. demonstrated that *Shigella* blocks secretion through the T3SS in anaerobic conditions through fumarate and nitrate reductase (FNR)-mediated suppression of *spa32* and *spa33* transcription (Marteyn et al., 2010). Detection of O_2_ in the region immediately adjacent to the epithelial barrier relieves this transcriptional suppression, triggering *spa32* and *spa33* expression leading to activation of the T3SS and efficient invasion (Marteyn et al., 2010). Altogether, these findings indicate that *Shigella* has evolved to acutely sense when it is in the appropriate gut environment to trigger increased adherence and T3SS activity.

At the distal end of the T3SS is the tip complex composed of IpaB, IpaC, and IpaD (Veenendaal et al., 2007). IpaD facilitates assembly of IpaB and IpaC onto the needle, and IpaB and IpaC are hydrophobic proteins that are able to insert into eukaryotic membranes to form a pore that allows for effector delivery (Blocker et al., 1999; Veenendaal et al., 2007). Membrane insertion and T3SS activity is promoted by interaction of IpaB with cholesterol in the host membrane (Hayward et al., 2005; Epler et al., 2009). Prior to insertion, IpaB functions to block secretion through the T3SS, acting as a molecular plug that is removed with insertion into the host membrane (Roehrich et al., 2010).

T3SS-dependent delivery of effector proteins generates a region of actin remodeling and polymerization that ultimately leads to membrane ruffling and the uptake of the associated *Shigella*. The effectors IpgB1 and IpgB2 act as guanine nucleotide exchange factors (GEFs) for Rac and RhoA to drive actin remodeling (Huang et al., 2009). Indeed, a *Shigella* double mutant for IpgB1 and IpgB2 is unable to efficiently invade intestinal epithelial cells (Hachani et al., 2008). In addition, *Shigella* IpgD is a phosphatase that generates phosphotidyl-inositol-5-phosphate to promote actin polymerization (Pendaries et al., 2006). The tip complex protein, IpaC, promotes invasion by activating Src kinase and Cdc42 (Mounier et al., 2009). Collectively, these events generate a region of intense actin remodeling. Finally, during the effector-induced membrane ruffles, IpaA binds to host-cell vinculin, a component of focal adhesions. Interaction with vinculin functions to anchor *Shigella* to the site of membrane ruffling (Izard et al., 2006).

Once *Shigella* is internalized, it rapidly escapes from the entry vacuole. IpaB and IpaC insertion into the vacuole membrane is thought to form holes promoting vacuole breakdown. More recent work identified that the recruitment of the host cell recycling endosome machinery by IpgD was critical for the rapid escape from the entry vacuole (Mellouk et al., 2014). Mellouk et al. demonstrated that IpgD phosphatase activity is required to drive recruitment of Rab11-positive recycling endosomes to the entry vacuole; however, the exact mechanism through which recycling endosomes promote vacuole escape requires further examination (Mellouk et al., 2014).

## Impact of *Shigella* invasion on metabolic pathways of the host and the pathogen

**Figure 2:**
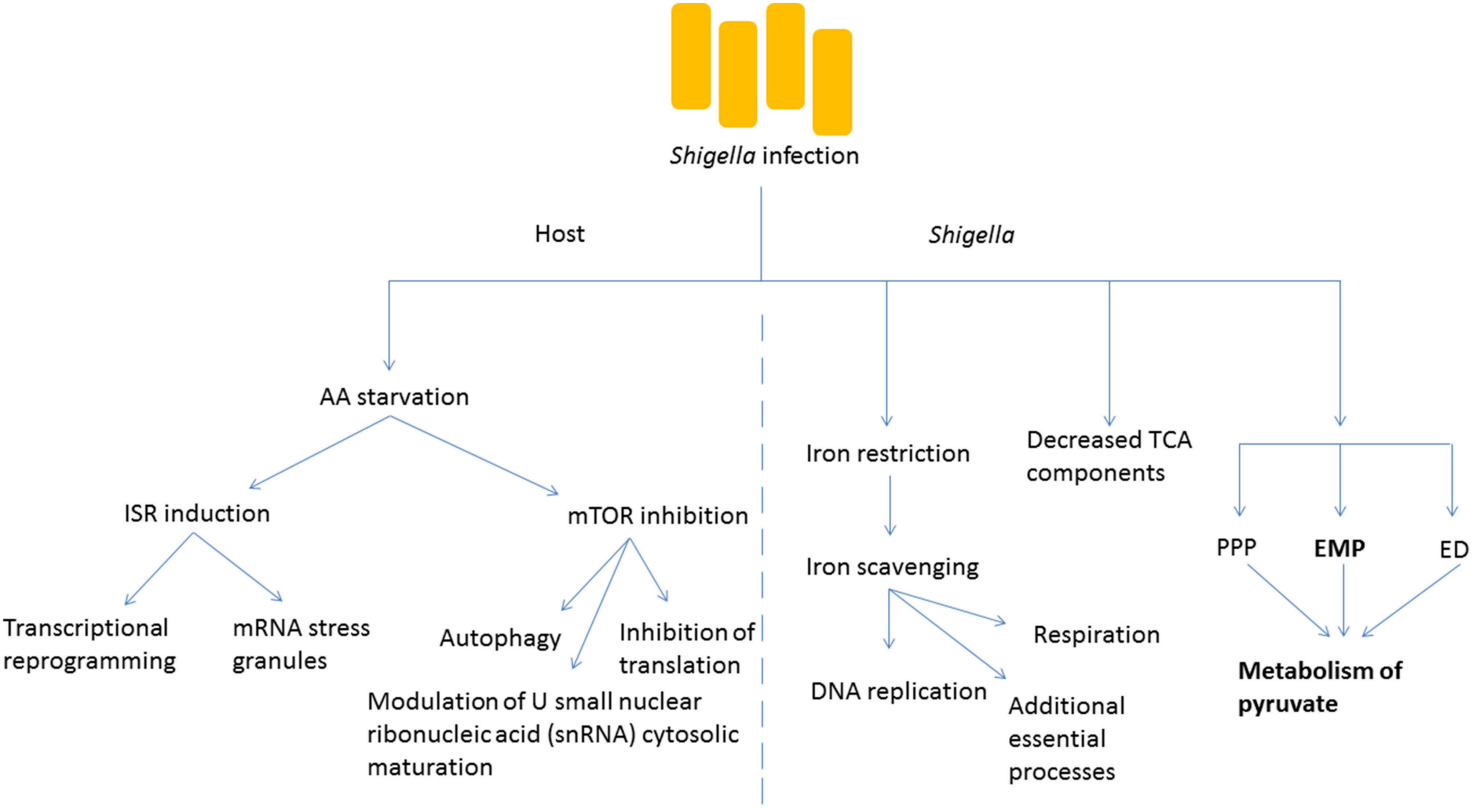
***Shigella* infection induces metabolic changes in the host and the bacteria**. Amino acid (AA) starvation is induced following bacterial infection, and this leads to numerous changes in the metabolism of the host cell including the induction of the integrated stress response (ISR) causing transcriptional reprogramming and formation of stress granules. The inhibition of mTOR signaling is also seen, with multiple downstream effects. Within *Shigella*, key cofactors such as iron need to be scavenged for essential survival processes. The tricarboxylic acid (TCA) cycle is less important than other processes such as fermentation. The Embden-Meyerhof-Parnas (EMP) pathway predominates the generation of pyruvate in comparison to the pentose-phosphate pathway (PPP) and the Entner-Doudoroff (ED) pathway.

The invasion of host cells by *Shigella* requires a dramatic adaptation of the pathogen to a new environment, in which access to a number of nutrients or metabolic cofactors, such as carbon sources, iron and oxygen is limited and is under the control of the host (Figure 2). Iron restriction is among the most well studied examples of nutrient stress that *Shigella* encounters as it grows inside host cells. While the host has developed multiple strategies, including the expression of iron-binding proteins, to limit the concentration of free iron inside cells because of its inherent toxicity, *Shigella* expresses molecules such as siderophores, heme transporters as well as ferric and ferrous iron transport systems that can capture intracellular iron (Payne et al., 2006). Recently, global proteomic analysis of *Shigella* inside host cells emphasized the importance of iron starvation stress in the intracellular milieu (Pieper et al., 2013). In particular, iron acquisition systems (Iut, Sit, FhuA, and Feo) and the iron starvation, stress-associated Suf protein are strongly upregulated in intracellular *Shigella*. In agreement, a global transcriptional study of *Shigella* growing inside host cells revealed that the bacteria experienced restricted levels of iron, magnesium and phosphate (Lucchini et al., 2005). Once properly scavenged, *Shigella* use iron for a number of essential processes ranging from DNA replication to respiration (Wei and Murphy, 2016). Moreover, progress has been made with regards to the intracellular source of carbon required by *Shigella* for its biosynthetic pathways. The global proteome analysis mentioned above also identified that mixed-acid fermentation, and metabolism of pyruvate in particular, is required for optimal intracellular growth of *S. flexneri* (Pieper et al., 2013). In contrast to fermentation pathway enzymes, components of the tricarboxylic acid (TCA) cycle were decreased in the intracellular bacteria, consistent with the notion that fermentation, rather than respiration, predominates in the intracellular environment. *Shigella* uses three glycolytic pathways to produce pyruvate from glucose: the Embden-Meyerhof-Parnas (EMP) pathway, the pentose-phosphate pathway (PPP) and the Entner-Doudoroff (ED) pathway. Studies by Payne's group demonstrated the importance of the EMP pathway, which converts glucose to pyruvate through the glucose-6-phosphate/fructose-6-phosphate/glyceraldehyde-3-phosphate intermediates, while the PPP and ED pathways were dispensable for *Shigella* intracellular growth (Waligora et al., 2014). Importantly, growth defects caused by glycolysis mutants could be overcome by supplementation with pyruvate, showing that this molecule is an essential carbon source for *Shigella* intracellular growth. In a complementary study, Kentner et al. analyzed host fermentation pathways in uninfected vs. *Shigella*-infected cells (Kentner et al., 2014). They observed that host glycolytic pathways were not substantially affected by the pathogen, but infected cells excreted acetate instead of pyruvate and lactate, suggesting that the latter molecules had been captured and used by *Shigella* for its metabolism. Importantly, this study also showed that pyruvate was the most important metabolite required for *Shigella* intracellular growth, while it appeared that access to host amino acids or fatty acids was not limiting for intracellular bacterial growth. This finding may reflect the landscape through which *Shigella* evolved over time, where amino acids and fatty acids were more limited, and access was more difficult than for attaining other metabolites.

While intracellular *Shigella* experience metabolic and nutrient stress, the infection also has major consequences for the infected host cell in terms of metabolic stress pathway induction. The mechanistic target of rapamycin (mTOR) and integrated stress response (ISR) pathways represent two major signaling axes in eukaryotic cells that control metabolic stress responses to nutrient limitation. We recently demonstrated that infection by *Shigella* resulted in potent inhibition of mTOR signaling, as well as induction of the ISR, and that the underlying mechanism was the induction of an amino acid (AA) starvation response in infected host cells (Tattoli et al., 2012). This AA starvation response upregulated the general control nonderepressible (GCN2)/eukaryotic translation initiation factor 2 (eIF2α)/activating transcription factor 3 (ATF3) axis of the ISR, resulting in stress-associated transcriptional reprogramming and accumulation of messenger ribonucleic acid (mRNA) stress granules in infected cells. Inhibition of the mTOR signaling axis caused dephosphorylation of 4E-binding protein 1 (4E-BP1) and S6 kinase 1 (S6K1), general inhibition of translation and upregulation of autophagy (Tattoli et al., 2012). It is likely that the down-regulation of mTOR signaling in infected host cells is an important step in innate immune defense against *Shigella*, since autophagy against intracellular pathogens (also known as xenophagy) is critical for host defense. Interestingly, Lu et al. recently demonstrated that *Shigella* used the effector protein OspB to inhibit this process in the first phase of the infection by turning on mTOR complex 1 through the scaffolding protein IQ motif-containing guanosine triphosphate (GTP)ase-activating protein 1 (IQGAP1; Lu et al., 2015).

An unexpected consequence of the induction of metabolic stress pathways following *Shigella* infection was reported. Specifically, we observed in *Shigella-*infected cells the modulation of U small nuclear ribonucleic acid (snRNA) cytosolic maturation, which is a critical step in the control of the assembly of the spliceosome, the molecular machinery responsible for mRNA splicing (Tsalikis et al., 2015). We demonstrated that this effect required bacterial invasion and could be recapitulated by AA starvation, endoplasmic reticulum stress or mTOR inhibition (Tsalikis et al., 2015). While it is likely that the adaptation of the spliceosome metabolism during infection mirrors translation inhibition and is an energy conservation mechanism, the results obtained in this study suggest that this process plays a role in the overall regulation of stress- and inflammation-associated responses observed during infection. It remains to be determined if the regulation of the U snRNA levels in infected cells also contributes to shaping alternative splicing programs in infected cells, and if this potential mechanism is important for host defense.

## Host detection of *Shigella*

The host detects invasive pathogens through both intracellular and extracellular sensing mechanisms. Among others, the Toll-like receptors (TLRs) sense the extracellular and endosomal environment and drive proinflammatory signaling in response to specific stimulation. This TLR-based detection plays a major role in the response to *Shigella* by myeloid cells (Girardin et al., 2001). For example, it is well documented that detection of extracellular lipopolysaccharide (LPS) by TLR4 leads to an inflammatory response in myeloid cells (Poltorak et al., 1998; Hoshino et al., 1999). However, the intracellular detection of *Shigella* in epithelial cells has proven to be critical for the host response. The Nod-like receptors NOD1 and NOD2 respond to *Shigella* peptidoglycan (PGN) and control both mitogen-activated protein kinase (MAPK) and nuclear factor κB (NF-κB) proinflammatory signaling (Girardin et al., 2001, 2003a,b,c; Chamaillard et al., 2003; Inohara et al., 2003). Studies have shown that NOD1 is essential for the induction of chemokines and cytokines in response to either intracellular *Shigella* or PGN. Bacterial replication inside the host cells releases PGN, which triggers the NOD1-receptor-interacting protein 2 (RIP2)-inhibitor of nuclear factor kappa-B kinase (IKK) pathway leading to the production of interleukin 8 (IL-8; Girardin et al., 2001). NOD1 remains the primary intracellular detector of *Shigella* infection, and is upstream of inflammatory as well as survival pathways that help the host handle infection by the pathogen. One such survival pathway involving NOD1-RIP2-IKKβ-NF-κB in epithelial cells counters the mitochondrial damage and stress pathway triggered by *Shigella* (Carneiro et al., 2009). The signaling pathways downstream of these receptors can function in isolation or in combination, leading to anti-microbial responses. For example, TLR stimulation can aid in NLR driven processes, as seen with the TLR-driven expression of the precursors of IL-18 and IL-1β, which are processed intracellularly as a consequence of NLR-dependent activation of inflammasomes (Miao et al., 2010). This allows for additional levels of amplification, ensuring a rapid response is initiated once a potential threat is verified through multiple sensors, both intracellularly and extracellularly.

After macrophages internalize *Shigella*, a proinflammatory, caspase-dependent form of cell death known as pyroptosis is induced by bacterial components as well as downstream danger signals coming from dead or dying host cells. The rod protein MxiI and the needle protein MxiH of *Shigella* T3SS have been shown to stimulate the NLR family CARD domain containing 4 protein (NLRC4) inflammasome after being sensed by NLR family, apoptosis inhibitory protein 2 (NAIP2) and human NAIP (murine NAIP1) respectively (Yang et al., 2013; Suzuki et al., 2014). The canonical NLR family, pyrin domain containing 3 (NLRP3) inflammasome is known to respond to phagocytic membrane remnants, among other stimuli (Ashida et al., 2014). Taken together, the presence of an intracellular system that generates specificity through different upstream sensors allows a few key effectors to carry out the same function while detecting a wide diversity of stimuli. However, the discovery of a host method for sensing intracellular LPS has uncovered the existence of a noncanonical inflammasome. This noncanonical inflammasome pathway requires caspase-11 (mouse homolog of human caspase-4) as an upstream sensor of intracellular LPS (Kayagaki et al., 2013). Caspase-11 induces pyroptosis independently of NLRP3 or caspase-1, but requires the latter components to induce the IL-1β and IL-18 cytokine response (Kayagaki et al., 2011). The discovery of this caspase-11 function changed the belief that LPS was only sensed extracellularly, by TLR4. A redundant system for sensing LPS reinforces the reliance on both intracellular and extracellular sensors for bacterial components, which together may serve to induce multiple waves of host defense. Although these findings concerning caspase-11 are not *Shigella-*specific, they suggest a mechanism that may be at play in cells in response to *Shigella* as well as other pathogens.

As the pathways for sensing intracellular viral nucleic acids have become more comprehensive, investigation into the intracellular sensors of bacterial nucleic acid has become an area of interest. Studies have reported that the intracellular detector of double-stranded deoxyribonucleic acid (dsDNA) is cyclic GMP-AMP synthase (cGAS), an enzyme that generates cyclic guanosine monophosphate-adenosine monophosphate (cGAMP) which then serves as a downstream second messenger to activate stimulator of interferon genes (STING) leading to the generation of type-1 interferons and NF-κB signaling (Broz and Monack, 2013; Wu et al., 2013). Cytosolic dsDNA associated with Shigella infection could arise from host cell damage or the presence of intracellular bacteria, both being situations where the host would benefit from proinflammatory signaling. While host cells independently possess multiple ways to respond to *Shigella* infection, recent studies highlight the intercellular transport of cGAMP via gap junctions. This transferred cGAMP was shown to stimulate STING in non-infected cells adjacent to the infected cell, allowing proactive defense preparation including proinflammatory cytokine expression in non-infected host cells (Kasper et al., 2010; Ablasser et al., 2013). Considering the bacterium rapidly moves from one cell to an adjacent cell using cell-to-cell spread mechanisms, any form of preparation that surrounding cells can take to preemptively raise a defense would be beneficial to combat *Shigella* infection (see below).

## *Shigella* effectors and manipulation of host processes

*Shigella* has evolved sophisticated strategies to escape the entry vacuole and to replicate in the host's intracellular compartment. These strategies depend on protein effectors which together greatly contribute to the manipulation of key cellular processes by the bacterial pathogen (Table 1). The effectors delivered by *Shigella* via the T3SS can be split up into a first and second wave. The first wave of effectors, as mentioned above, are essential for actin remodeling and subsequent invasion into host epithelial cells, and include proteins such as IpaA, IpaB, IpaC and VirA (Wang et al., 2013). Once *Shigella* has entered the cell, the second wave of effectors causes changes in host membrane trafficking, autophagy, inflammatory and death signaling, epigenetic modifications, among other processes like intracellular motility and intercellular dissemination that are mentioned above (Ashida et al., 2015). These changes promote *Shigella* survival, replication and spread to surrounding cells, allowing the infection to worsen.

**Table 1:** ***Shigella* effectors manipulate a variety of host processes**.

**Summary of *Shigella* effectors and their mechanisms of action**		
***Shigella*** **effector**	**Host targets**	**Effect on host process**
OspE1	Bind to exterior of polarized cells	Increase adherence to polarized cells
OspE2	Bind to exterior of polarized cells	Increase adherence to polarized cells
IpgB1	Act as GEF for Rac	Induce actin remodeling to facilitate invasion
IpgB2	Act as GEF for RhoA	Induce actin remodeling to facilitate invasion
IpgD	Act as phosphatase to generate PI5P	Induce actin remodeling to facilitate invasion
IpaB	Bind to filopodia and cholesterol on the host cell surface Insert into vacuole membrane Reduce and disrupt balanced levels of cholesterol and lipids	Insert into eukaryotic membrane to form a pore Facilitate invasion Form T3SS complex Promote vacuole breakdown Interfere with proper Golgi function
IpaD	Bind to filopodia on the host cell surface	Facilitate interaction and invasion Form T3SS complex
IpaC	Activate Src Kinase and Cdc42 Insert into vacuole membrane	Facilitate invasion Form T3SS complex Promote vacuole breakdown
IpaA	Host-cell focal adhesion component vinculin	Functions as an anchor to the site of membrane ruffling
OspB	Induce mTOR signaling through IQGAP1	Inhibit autophagy
VirA	Catalyze GTP hydrolysis in Rab1 Induce p53 degradation	Disrupt ER-to-Golgi trafficking and autophagy Block apoptosis
IpgD	Host cell recycling endosome machinery Increase levels of PI5P leading to p53 degradation	Rapid escape from the entry vacuole Induce PI3K/Akt-dependent survival pathways
IpaJ	Cleave N-myristoylated glycine from ARF1 to disrupt its localization	Disrupt autophagosome maturation and host membrane trafficking
IpaH9.8	Target and degrade NEMO/IKKγ	Disrupt NF-κB signaling
IpaH4.5	Target and degrade p65 subunit of NF- κB	Disrupt NF- κB signaling
IpaH0722	Target and degrade TRAF2	Disrupt NF- κB signaling
OspZ	Prevent nuclear translocation of p65	Disrupt NF- κB signaling
OspI	Deamidate UBC13 E2 enzyme needed for activation of TRAF6	Disrupt NF- κB signaling
OspG	Interfere with ubiquitin proteasomal degradation of IκB-α	Disrupt NF- κB signaling
OspF	Inactivate MAPK signaling components like ERK and p38 by epigenetic modifications using its phosphothreonine lyase activity	Disrupt MAPK signaling
OspC3	Interact with caspase-4-p19 subunit and inhibit its heterodimerization and activation	Inhibit pyroptosis within epithelial cells
IcsB	Block autophagy targeting by binding Atg5 Bind cholesterol	Reduce autophagy Escape from the vacuole following cell-to-cell spread

*Such processes include apoptotic signaling in addition to other death signaling, inhibition of autophagy, actin remodeling which is used in invasion as well as motility and cell-to-cell spread, anti-inflammatory signaling leading to an altered immune response, altered membrane dynamics and the stimulation of inflammasome formation and action. These altered processes contribute to Shigella infection, aiding in efficient propagation of the bacteria within the host*.

As will be explained in detail below, host cells undergo autophagy in response to various stresses, one of them being infection (Baxt and Goldberg, 2014). *Shigella* deliver effectors similar to those involved in the first wave that target actin polymerization and motility to manipulate the host machinery involved in autophagy. Likewise, *Shigella* inject effectors that interfere with host membrane trafficking. IpgD is one such effector, and as a phosphoinositide phosphatase it promotes phosphatidylinositol 5-phosphate (PI5P) production, which plays numerous roles in the manipulation of host trafficking such as increasing vacuolar membrane disruption (Ashida et al., 2015). IpgD-driven PI5P also blocks hemichannels from forming, preventing the release of adenosine triphosphate (ATP), limiting the neighboring cells from sensing this potential danger signal (Puhar et al., 2013). Three other effectors, VirA, IpaJ and IpaB also aid in this manipulation of host membrane trafficking. GTPases form important components of the host secretory pathway, and are targeted by VirA and IpaJ. VirA disrupts ER-to-Golgi trafficking and autophagy by catalyzing GTP hydrolysis in Rab1, and IpaJ cleaves the N-myristoylated glycine from ARF1, disrupting localization and autophagosome maturation among other processes (Ashida et al., 2015). Lastly, IpaB interferes with proper Golgi functioning and protein transport through reducing, and therefore disrupting the balanced levels of cholesterol and lipids (Ashida et al., 2015). Thus, altering membrane dynamics appears to be a key component of the strategy of *Shigella* infection.

Inflammatory signaling is one of the fundamental host defenses to infection, making it a candidate for *Shigella* targetting. Indeed, *Shigella* dampens the NF-κB signaling pathway using a number of effectors, such as the IpaH ubiquitin ligase family. IpaH9.8 targets NF-κB essential modulator (NEMO)/IKKγ, IpaH4.5 targets p65 subunit of NF-κB, and IpaH0722 targets TNF Receptor-Associated Factor 2 (TRAF2), together degrading important components of the NF-κB pathway, halting pro inflammatory signaling (Ashida et al., 2013; Wang et al., 2013; Giogha et al., 2014). Likewise, the Osp family of effectors also contributes to NF-κB blockage, through different mechanisms. OspZ prevents the translocation of p65 to the nucleus, OspI deamidates the UBC13 E2 enzyme needed for activation of TRAF6, and OspG interferes with the ubiquitin proteasomal degradation of IκB-α.This multifaceted attack on NF- κB confirms its importance in *Shigella* clearance. Aside from NF- κB, *Shigella* effectors also target other signaling pathways that regulate inflammation. OspF uses its phosphothreonine lyase activity to irreversibly inactivate MAPK signaling components like extracellular signal-regulated kinase (ERK) and p38 through epigenetic modifications (Li et al., 2007; Newton et al., 2010; Sanada et al., 2012; Raymond et al., 2013). These methods of disruption lead to a decrease in proinflammatory, apoptotic and stress signaling gene expression in host cells.

As *Shigella* impose such a severe halt on host NF-κB signaling, it is reasonable to expect the host cell to become pro-apoptotic. However, *Shigella* do not induce cell death in epithelial cells as easily as they do in macrophages, and as a result, it is common to see epithelial cells with hundreds of bacteria in them (Mantis et al., 1996). As expected, *Shigella* inject effectors that target the apoptotic pathway as well. VirA and IpgD lead to p53 degradation through calpain activation and proteasomal degradation respectively, halting cell death. IpgD increases levels of PI5P which promote phosphoinositide 3-kinase (PI3K)/Akt-dependent survival pathways, and phosphorylation of mouse double minute 2 homolog (MDM2) which degrades p53 (Ashida et al., 2015). A survival process induced by VirA involves the calpain-driven cleavage of BH3 interacting domain death agonist (BID), which leads to second mitochondria-derived activator of caspases (SMAC) release from mitochondria and the blockage of X-linked inhibitor of apoptosis protein (XIAP)-driven immune response and apoptosis (Andree et al., 2014; Ashida et al., 2014). On a similar note, although *Shigella* induce pyroptosis in macrophages, recent studies have identified the *Shigella* effector OspC3 as an antagonist to the caspase-4-driven pyroptosis within epithelial cells (Kobayashi et al., 2013). This is in line with the priority of *Shigella* to keep host epithelial cells alive, which they accomplish by injecting T3SS effectors.

## Cell-to-cell spread and autophagy targeting

In the cytosol, *Shigella* use the host cell actin machinery for intracellular motility. IcsA (VirG) interacts with and activates neuronal Wiskott-Aldrich Syndrome protein (N-WASP), which recruits Arp2/3 to drive actin polymerization (Egile et al., 1999). The tyrosine kinase, Btk, phosphorylates N-WASP, increasing Shigella motility (Egile et al., 1999; Dragoi et al., 2013). Importantly, actin-based motility enables *Shigella* to spread from cell-to-cell through the formation of protrusions in the cell membrane that preferentially occur at tricellular junctions (Fukumatsu et al., 2012; Figure 1). This process is facilitated by the host proteins Serine/Threonine Kinase 11 (STK11), Myosin X, Myosin II and Dia1 (Rathman et al., 2000; Heindl et al., 2010; Bishai et al., 2013; Dragoi and Agaisse, 2014). Importantly, recent work has demonstrated that T3SS secretion is suppressed during intracellular replication but is reactivated during cell-cell spread (Campbell-Valois et al., 2014). In agreement, using a system that allows for the post-entry shutdown of T3SS activity, Kuehl et al. demonstrated that cell to cell spread is dependent on T3SS activity (Kuehl et al., 2014).

An important challenge *Shigella* must overcome during intracellular replication is the avoidance of autophagy targeting and degradation. Autophagy is a complex system for sequestering cytoplasmic cargo in *de novo* generated double membrane vesicles that subsequently fuse with lysosomes to allow for degradation of the cytoplasmic cargo. Although the primary function of autophagy is the recycling of cytoplasmic components or entire organelles, it can also be used as a cellular defense pathway by targeting intracellular bacteria. Ogawa et al. reported the first interaction of intracellular *Shigella* with the autophagy machinery (Ogawa et al., 2005). In this initial report, the authors demonstrated that the surface protein IcsB was critical in reducing autophagy targeting and that strains lacking IcsB are efficiently restricted by autophagy (Ogawa et al., 2005). Interestingly, strains lacking IcsA (VirG) and IcsB are not targeted by autophagy (Ogawa et al., 2005). Subsequent work demonstrated that Tecpr1 is a host targeting factor in this pathway (Ogawa et al., 2011). These findings provided the first suggestion that the machinery involved in cell-cell spread and intracellular motility is linked with autophagy targeting.

A number of studies have suggested that *Shigella* is particularly vulnerable to autophagy targeting when it is associated with host cell membranes. Travassos et al. demonstrated that the intracellular peptidoglycan pattern recognition receptors (PRRs), NOD1 and NOD2, play a critical role in targeting the autophagy machinery through interaction with autophagy related 16 like 1 (ATG16L1) to the site of *Shigella* invasion at the plasma membrane (Travassos et al., 2010). This NOD-ATG16L1 interaction also regulates the proinflammatory response triggered by *Shigella* invasion (Sorbara et al., 2013). Furthermore, the host membrane remnants resulting from the escape of *Shigella* from the entry vacuole can drive inflammatory responses, and are also ubiquitinated and targeted for autophagic degradation (Dupont et al., 2009). Mostowy et al. found that intracellular *Shigella* can be trapped in Septin cages allowing for autophagy targeting and removal. In contrast, bacteria with actin tails are not Septin-caged or autophagy-targeted (Mostowy et al., 2010).

Work from both Ogawa et al. and Mostowy et al. suggest that components involved in actin based motility are necessary for both autophagy targeting (IcsA), and escape (IcsB; Ogawa et al., 2005; Mostowy et al., 2010). Recent work has shed light on these findings. Campbell-Valois et al. demonstrated that the cytosolic subset of *Shigella* during infection were not targeted to the autophagy system, and that only bacteria already associated with an entry vacuole or cell-cell spread event, as marked by an active T3SS, were targeted (Campbell-Valois et al., 2015). Furthermore, they found that IcsB and VirA are involved in escape from the vacuole following cell-to-cell spread, and that deficiency in either protein leads to increased LC3 targeting as a result of an impaired escape process (Campbell-Valois et al., 2015). In agreement, IcsA negative bacteria, deficient in cell-to-cell spread, are also not targeted by Septins or autophagy proteins (Ogawa et al., 2005, 2011; Mostowy et al., 2010). Although the exact mechanism through which IcsB promotes cell-to-cell spread is unknown, Kayath et al. demonstrated that cholesterol binding by IcsB is necessary for its function in autophagy avoidance (Kayath et al., 2010), suggesting that cholesterol binding during cell-to-cell spread contributes to escape. Together these findings are in agreement with a model in which *Shigella* is efficiently targeted to autophagy in the context of host membranes during either initial invasion or cell-to-cell spread.

## Conclusions

As is often the case with complex biological interactions between infectious agents and the host, the discovery of mechanistic insight and explanations uncovers additional questions. Some of those, together with brief conclusions are presented below.

The *in vivo* relevance of the autophagy targeting mechanism remains to be determined. In other words, does autophagy actually restrict growth of *Shigella* in physiological conditions? In addition to this, more work is needed to understand the molecular mechanism of how *Shigella* is able to destabilize membranes (either during initial invasion or cell-to-cell spread) and how that relates to host recognition of cytosolic *Shigella*.

While it is clear that infection with *Shigella* leads to the upregulation of metabolic stress pathways, it is unclear how the activation of these pathways contributes to host defense against this pathogen. Similarly, it remains to be seen how the upregulated stress pathway affects the transcriptional landscape of *Shigella*-infected cells. It has previously been shown that pyruvate is a major carbon source for intracellular *Shigella*, however these studies have been performed using cancer cells that extensively rely on glycolysis. To advance the understanding further, it will be essential to determine if the same holds true for primary intestinal epithelial cells, which represent a physiological target of *Shigella*. *Shigella* infection induces mTOR inhibition and eIF2alpha phosphorylation, but the extent to which these pathways are regulated by specific *Shigella* effectors remains poorly characterized. Identifying if, in addition to OspB, *Shigella* has evolved means to manipulate these pathways will be an important question for future investigations. Lastly, it was proposed that *Shigella* may induce amino acid starvation in host cells as a result of membrane damage, however, it currently remains unclear how host membrane damage triggers amino acid starvation.

*Shigella* effectors manipulate a variety of fundamental processes in the host. The list of *Shigella* effectors is still growing, along with the host processes that are targeted. The induction of cell death remains one of the fundamental defense mechanisms for host cells to combat infection. Further investigation into the risk/benefit of pyroptosis for *Shigella* will help understand which situations call for such a proinflammatory method of escape from the host cell. Discovery of the noncanonical inflammasome has raised interest concerning the exact nature of stimuli and sensors used by the major inflammasomes, so research focused on clarifying these pathways is needed. Finally, the identification of the intercellular transfer of protective messengers like cGAMP suggests that other paracrine messengers of infection may also exist. This could represent an effective, proactive defense strategy used by the host to counteract the deleterious consequence of the injection of effectors during invasion by alerting non-infected, neighboring cells.

Such progress in uncovering the pathogenesis of *Shigella* infection suggests possibilities for the future of therapies and treatment for diseases of a bacterial nature. Perhaps the most apparent treatment strategy would be designing specific inhibitors of the T3SS effectors, to block the action of those effectors that are critical to invasion and cell-to-cell spread. Likewise, after understanding the metabolic changes *Shigella* undergo during the infection process, limiting access to pyruvate could halt propagation of the bacteria. However, with most therapies that target specific components or processes of a microbe, mutations and selection leading to resistance would be a potential pitfall. With this in mind, an alternative route therapies could take involve strengthening the host responses, especially those that are targeted by the bacterial infection, before the bacteria have a chance to target the cells. This defense happens endogenously as surrounding cells amplify their defense when a neighbor is infected, therefore should also work with exogenous stimuli. Such avenues could include sustaining components of the inflammatory and survival signaling pathways, or limiting bacterial access to essential nutrients like iron in patients who have been infected. Each direction will benefit from further investigation into the molecular mechanisms behind *Shigella* pathogenesis, and the breadth of host response to infection.

## Author contributions

All authors listed, have made substantial, direct and intellectual contribution to the work, and approved it for publication.

### Conflict of interest statement

The authors declare that the research was conducted in the absence of any commercial or financial relationships that could be construed as a potential conflict of interest.
